# Diagnosis and treatment of chronic insomnia

**DOI:** 10.4103/0972-2327.64628

**Published:** 2010

**Authors:** Sahoo Saddichha

**Affiliations:** Department of Psychiatry, National Institute of Mental Health and Neurosciences, Bangalore, India

**Keywords:** Chronic, diagnosis, insomnia, treatment

## Abstract

Insomnia is a disorder characterized by inability to sleep or a total lack of sleep, prevalence of which ranges from 10 to 15% among the general population with increased rates seen among older ages, female gender, White population and presence of medical or psychiatric illness. Yet this condition is still under-recognized, under-diagnosed, and under-treated. This article aims to review the operational definitions and management of chronic insomnia. A computerized search on PubMed carried from 1980 to January 2009 led to the summarization of the results. There are several strategies to manage chronic insomnia. To initiate treatment, it is necessary to define it and differentiate it from other co-morbid psychiatric disorders. Non-pharmacologic strategies such as stimulus control therapy and relaxation and cognitive therapies have the best effect sizes followed by sleep restriction, paradoxical intention and sleep hygiene education which have modest to less than modest effect sizes. Among pharmacotherapeutic agents, non-benzodiazepine hypnotics are the first line of management followed by benzodiazepines, amitryptiline and antihistaminics. However, adequate trials of combined behavior therapy and pharmacotherapy are the best course of management.

Oh sleep, Oh gentle sleep,

Nature's soft nurse How have I frighted thee?

That though no more will weigh mine eyelids down

And sleep my senses in forgetfulness?

***Henry IV, William Shakespeare***

## Introduction

The word "insomnia' originates from the Latin "in" (no) and "somnus" (sleep). It is a disorder characterized by inability to sleep or a total lack of sleep. Being the first psychosomatic disorder to be described by Johann Heinroth in 1818, insomnia clinically presents as a subjective perception of dissatisfaction with the amount and/or quality of the sleep.[1] The presenting complaints are often that of[2] difficulties falling asleep in spite of being in bed, waking up often during the night and having trouble going back to sleep, waking up too early in the morning or having an unrefreshing sleep.

Various studies have noted insomnia to be quite a common condition with symptoms present in about 33–50% of the adult population.[3] The prevalence, however, ranges from 10 to 15% among the general population,[4] with higher rates seen among divorced, separated, or widowed people,[5] older ages, female gender,[6] White population,[7] and in the presence of co-morbid medical or psychiatric illness.[8] About 30% of all adults complain of occasional insomnia and 10% of chronic insomnia, of whom 40% may have a psychiatric illness.[9,10] Despite these high prevalence rates, evidence suggests that insomnia is mostly under-recognized, under-diagnosed, and under-treated,[4] with the condition continuing to remain persistent in 50–85% of individuals over follow-up intervals of one to several years.[11]

Chronic insomnia represents a more complex condition than acute transient insomnia. Patients with chronic insomnia usually have accompanying daytime impairment of cognition, mood, or performance that impacts not only the patient and his family, but also affects friends, coworkers, and caretakers. Insomnia patients are more likely to visit hospitals and physicians, have increased absenteeism, make errors or have accidents at work, and have more fatal road accidents.[12,13] There is also an increased risk for depression, anxiety, substance -use, suicide, and possible immune dysfunction.[14] It is imperative that clinicians remain alert to these possible individual and societal risks during the evaluation.

## Materials and Methods

The author carried out a computerized search on PubMed/MEDLINE for all articles in English published from 1980 to January 2009, using combinations of the following words: insomnia, chronic, sleep disorder, treatment, resistance, management, behavioral therapy, cognitive therapy, pharmacologic therapy, drugs. Subsequently, we searched the bibliographies of the articles selected via the first strategy. Full-text articles were retrieved with the help of institutional online access and by writing personally to the authors. The search led to 422 articles that pertained to chronic insomnia. Out of these, about 282 articles were retrieved using the above strategies. Assessment of this literature led to the identification of pertinent articles, which were then weighted according to a rating scheme based on levels of evidence according to NICE guidelines.[15] This was as follows:

Level Ia: evidence obtained from meta-analysis of randomized controlled trials;
Level Ib: evidence obtained from at least one randomized controlled trial;
Level IIa: evidence obtained from at least one well-designed controlled study without randomization;
Level IIb: evidence obtained from at least one other type of well-designed quasi-experimental study;
Level III: evidence obtained from well-designed nonexperimental descriptive studies, such as comparative studies, correlation studies, and case studies;
Level IV: evidence obtained from expert committee reports or opinions and/or clinical experiences of respected authorities.

These levels were further divided into grades which were

Grade A (evidence levels Ia, Ib): requires at least one randomized controlled trial as part of the body of literature of overall good quality and consistency addressing the specific recommendation;

Grade B (evidence levels IIa, IIb, III): requires availability of well-conducted clinical studies but no randomized clinical trials on the topic of recommendation;

Grade C (evidence level IV): requires evidence obtained from expert committee reports or opinions and/or clinical experiences of respected authorities. Indicates absence of directly applicable clinical studies of good quality.

Good practice points (GPP): recommended best practice based on the clinical experience of the guideline development group.

The author then evaluated the articles in a non-quantitative manner, collated them, and summarized the results.

## Results and Discussion

### Definition of chronic insomnia

Although there are various definitions of chronic insomnia, the most widely accepted[16] is the one that defines it to be a condition characterized by "inadequate quantity or quality of sleep characterized by a subjective report of difficulty with sleep initiation, duration, consolidation, or quality that occurs despite adequate opportunity for sleep, and that results in some form of daytime impairment and has persisted for at least one month". The causes of this are many, the most important of which are medications, drug or alcohol abuse, psychiatric disorders like depression or anxiety, medical disorders (such as arthritis, asthma, Parkinson's disease, hyperthyroidism, prostate hypertrophy, degenerative neurological disorders, renal disorders, heart failure, rhinitis), poor sleep hygiene, and other disorders like sleep apnea, periodic limb movements, conditioned insomnia (behavioral conditioning), restless legs syndrome, circadian rhythm disorder or advanced/delayed sleep-phase syndrome. Neurological causes such as fibromyalgia and Morvan's syndrome, medical causes such as gastroesophageal reflux disease, and in children, sleep-onset association disorder and limit-setting sleep disorder also need to be identified and addressed since these can commonly present as chronic insomnia.

Difficulty in falling asleep may be primarily due to behavioral and cognitive factors such as worrying in bed or having unreasonable expectations of sleep duration.[17] Gillin[18] believes that this excessive worry about sleep loss eventually becomes persistent and "provides an automatic nocturnal trigger for anxiety and arousal." Further, unsuccessful attempts to control thoughts, images, and emotions only worsen the situation. After such a cycle is established, "insomnia becomes a self-fulfilling prophecy that can persist indefinitely." Other behaviors in bed or in the bedroom that are incompatible with sleep may include talking on the telephone at night, watching television, using computers, exercising, eating, smoking, or "clock watching."

### Evaluation of a patient with insomnia

As insomnia is both a symptom and a disorder in itself, detailed evaluation of the problem is imperative before reaching a clinical diagnosis. The treating clinician should have a high index of suspicion of insomnia or sleep difficulty when patients present with the following symptoms:[19] fatigue, excessive daytime sleepiness, major and/or minor depressive episode, generalized anxiety disorder, memory/concentration complaints, pain.

The mandatory assessment of insomnia includes the following.

#### Sleep history

Sleep history is the first step in evaluation of primary insomnia, which provides the clinician with a structured approach to a diagnosis. It requires a general description of the disorder, i.e., its duration, severity, variation, and daytime consequences. The NHLBI Working Group has devised the following approach which may be followed [Table 1].[20]

**Table 1:** Evaluation of insomnia

Primary area of focus	Sample questions
What is the nature and severity of the problem?	Do you have diffi culty primarily in
	falling asleep
	staying asleep
	waking too early
	Do you have trouble going back to sleep if you wake during the night?
	Do you take any drugs/medications to help you sleep?
	What are the day time consequences of your sleep problem? (e.g., fatigue, irritability, difficulty in concentration etc.)
	How many nights per week/month does your insomnia occur? Is it related to season, menstrual cycle or any other cyclical factors?
Is the patient's environment disturbing?	Is there anything in your home that disturbs your sleep such as loud TV, pets, infants, noise, lights, etc.?
What is the patient's sleep routine?	At what time do you get into bed and try to sleep?
	At what time do you get up in the morning?
	How many hours in the night do you actually sleep (out of total time spent in bed)?
	Is your occupation timings causing the sleep problems? (work schedule, shift duty, jet lag etc.)
	Do you sleep during the day or evening?
Identify maladaptive behaviors	Do you consume nicotine, tea/coffee, or alcohol prior to sleep?
	What do you do each night before going to bed?
	When you wake up in the night, do you eat/smoke/check the clock?

#### Use of prescription drugs

Various prescription drugs may be responsible for chronic insomnia. Such a use should be asked for specifically and ruled out. The drugs may include anticonvulsants such as phenytoin and lamotrigine, beta-blockers like acebutolol, atenolol, metoprolol, oxprenolol, propranolol, and sotalol, antipsychotics like sulpiride, antidepressants such as Selective Serotonin Reuptake Inhibitors (SSRIs) or Monoamine oxidase inhibitors (MAOIs) and non-steroidal anti-inflammatory drugs (NSAIDs) such as indomethacin, diclofenac, naproxen, and sulindac.

#### Sleep diary or sleep log

A sleep diary helps in specifically estimating the severity of the problem, the night to night variability, and presence of maladaptive habits such as taking naps or spending excessive time in bed (more than 8 hours). Sleep diary also keeps track of compliance with behavioral interventions and response to treatment.

#### Sleep and psychological rating scale

Epworth Sleepiness Scale (ESS) rates the chance of dozing in the following situations[21] which may be during sitting and reading, watching television, sitting inactively in a public place, being a passenger in a car for an hour without a break, during lying down to rest in the afternoon, sitting and talking to someone, sitting quietly after lunch without alcohol or while waiting at a traffic signal in a car.

The ESS is rated on a 4-point scale for each of the above factors based on the following scores:

0 – no chances of dozing;
1 – slight chances of dozing;
2 – moderate chances of dozing; and
3 – high chances of dozing.

A score of more than 16 indicates daytime somnolence, while a cutoff of 11 is often employed to indicate a possible disorder associated with excessive sleepiness.

#### Focused physical examination

A general physical examination may help assess certain organic pathologies such as chronic obstructive pulmonary diseases (COPD), asthma, or restless leg syndrome which may disturb sleep.

#### Blood tests

Blood tests may help to rule out subtle manifestations of thyroid diseases, iron deficiency anemia, and vitamin B12 deficiency (restless leg syndrome).

#### Polysomnography

It is considered the gold standard for measuring sleep. electroencephalogram (EEG), electrooculography (EOG), electromyography (EMG), electrocardiography (ECG), pulse oximetry, and air flow are used to reveal a variety of findings like periodic limb movement disorder, sleep apnea, and narcolepsy.[22]

#### Actigraphy

Actigraphy measures physical activity with a portable device (usually including an accelerometer) worn on the wrist. Data recorded can be stored for weeks and then downloaded into a computer. Sleep and wake time can be analyzed by analyzing the movement data. This approach to estimating sleep and wake time has been shown to correlate with polysomnographic measures in normal sleepers, with reduced values noted in patients with insomnia.[2,23]

#### Summary of investigations

Investigations do not always correlate well with the patient's experience of insomnia and cannot replace a thorough clinical evaluation. Hence, it is important to recognize that insomnia is a subjective clinical diagnosis, and therefore, a patient's subjective report of sleep difficulties should play the most important role in directing management in most cases. It is also important to ask questions about the range of symptoms experienced and changes over time. Because insomnia is a patient-reported symptom, rather than a polysomnographically defined disorder, referral to a sleep laboratory for polysomnographic diagnosis should be reserved for cases in which another primary sleep disorder, such as obstructive sleep apnea or periodic movement disorder, is suspected, because these may require greater expertise in sleep medicine.[24] Other measures that can be used are evaluation of mental status, subjective sleep quality, psychological assessment scales, daytime function, quality of life, and dysfunctional beliefs and attitudes.

#### Diagnosis

The DSM IV TR [Table 2][25] provides a list of diagnostic criteria for primary insomnia. The term "primary" indicates that the insomnia is independent of any known physical or mental condition.

**Table 2:** Diagnosis of primary insomnia

DSM IV TR criteria of primary insomnia
These include any of the following:
The predominant complaint is difficulty initiating or maintaining sleep, or non-restorative sleep, for at least 1 month. The sleep disturbance (or associated daytime fatigue) causes clinically significant distress or impairment in social, occupational, or other important areas of functioning The sleep disturbance does not occur exclusively during the course of narcolepsy, breathing-related sleep disorder, a circadian rhythm sleep disorder or a parasomnia. The disturbance does not occur exclusively during the course of another mental disorder (e.g., major depressive disorder, generalized anxiety disorder, a delirium). The disturbance is not due to the direct physiological effects of a substance (e.g., a drug of abuse, a medication) or a general medical condition.

The International Classification of Sleep Disorders 2[26] codes insomnia under the broad heading of dyssomnias, either intrinsic or extrinsic sleep disorders. Based on the severity, it classifies insomnia into three types as follows.

1. Mild insomnia:This term describes an almost nightly complaint of an insufficient amount of sleep or not feeling rested after the habitual sleep episode. It is accompanied by little or no evidence of impairment of social or occupational functioning. Mild insomnia is often associated with feelings of restlessness, irritability, mild anxiety, daytime fatigue, and tiredness.
2. Moderate insomnia: This term describes a nightly complaint of an insufficient amount of sleep or not feeling rested after the habitual sleep episode. It is accompanied by mild or moderate impairment of social or occupational functioning. Moderate insomnia is always associated with feelings of restlessness, irritability, anxiety, daytime fatigue, and tiredness.
3. Severe insomnia: This term describes a nightly complaint of an insufficient amount of sleep or not feeling rested after the habitual sleep episode. It is accompanied by severe impairment of social or occupational functioning. Severe insomnia is associated with feelings of restlessness, irritability, anxiety, daytime fatigue, and tiredness.

### Treatment of Chronic Insomnia

The treatment of chronic insomnia consists of initially diagnosing and treating the underlying medical or psychological problems. The identification of behaviors that may worsen insomnia follows and stopping (or reducing) them would help eliminate insomnia. Next, a possible trial of pharmacology can be tried, although the long-term use of drugs for chronic insomnia is controversial. This is in spite of the fact that the US FDA has approved three medications for the treatment of insomnia with no limitation on the duration of their use. A trial of behavioral techniques to improve sleep, such as relaxation therapy, sleep restriction therapy, and reconditioning, is however useful. Behavioral intervention combined with pharmacologic agents may be more effective than either approach alone.

### Non-pharmacologic management strategies

Non-pharmacologic interventions for insomnia consist primarily of short-term cognitive-behavioral therapies. These methods act primarily by reducing heightened autonomic and cognitive arousal, modifying self-perpetuating maladaptive sleep habits, altering dysfunctional beliefs and attitudes about sleep, and educating patients about healthier sleep practices.[27] The techniques include the following.

#### Stimulus control therapy

Stimulus control therapy is based on the premise that insomnia is a conditioned response to temporal (bedtime) and environmental (bed/bedroom) cues that are usually associated with sleep.[28] Accordingly, the main objective of stimulus control therapy is to train the patient to "re-associate the bed and bedroom with rapid sleep onset by curtailing sleep-incompatible activities (overt and covert) that serve as cues for staying awake and by enforcing a consistent sleep-wake schedule." Stimulus control therapy consists of the following instructional procedures[29] consisting of going to bed only when feeling sleepy, using the bed and bedroom only for sleep and sex and nothing else like watching TV, getting out of bed and going into another room whenever unable to fall asleep or returning to sleep within 15–20 minutes and returning to bed only when sleepy again, maintaining a regular rising time in the morning regardless of sleep duration the previous night, and avoiding daytime napping.

Evidence suggests that stimulus control therapy is effective and well suited for the clinical management of insomnia in the elderly[30] with effect sizes ranging from 0.81 to 1.16 for sleep latency, 0.41 to 0.38 for total sleep time, and 0.70 for wake after sleep onset.[31,32]

#### Sleep restriction

Sleep restriction therapy consists of restricting the amount of time spent in bed to nearly match the subjective amount of time spent sleeping.[30] For example, if a person reports sleeping an average of 5 hours per night out of 8 hours spent in bed, the initial prescribed "sleep window" (i.e., from initial bedtime to final arising time) would be 5 hours. Subsequently, the allowable time in bed is increased by 15–20 minutes for a given week when sleep efficiency (defined as ratio of total sleep/time spent in bed × 100%) exceeds 90%, decreased by the same amount of time when sleep efficiency is lower than 80%, and kept stable when sleep efficiency falls between 80 and 90%. Periodic adjustments are made (usually on a weekly basis) until an optimal sleep duration is achieved. Sleep restriction therefore creates a mild state of sleep deprivation and is said to "promote a more rapid sleep onset, more efficient sleep, and less inter-night variability." To prevent excessive daytime sleepiness, time spent in bed should not be less than 5 hours per night. Evidence suggests that sleep restriction therapy is moderately effective with effect sizes ranging from 0.85 to 0.98 for sleep latency, –1.06 to 0.37 for total sleep time and 0.76 for wake after sleep onset.[31,34]

#### Relaxation therapies

Relaxation-based interventions are based on the observation that insomnia patients often display high levels of arousal (physiological and cognitive), both at night and during daytime.[33] Relaxation methods are used to deactivate the heightened arousal system, and the selection of a specific technique varies depending on whether physiological or cognitive arousal is targeted for treatment. *Progressive muscle relaxation* and *biofeedback* techniques seek to reduce somatic arousal, whereas attention focusing procedures such as *imagery training* and *thought stopping* are intended to lower presleep cognitive arousal (e.g., intrusive thoughts, racing mind). Additional relaxation therapies (e.g., abdominal breathing, meditation, hypnosis) have also been advocated, but currently there is no evidence to support their use in the clinical management of insomnia with less than modest effect sizes ranging from 0.81 to 0.83 for sleep latency, 0.25 to 0.52 for total sleep time, and 0.06 for wake after sleep onset.[31,32] As is the premise for most self-management skills, all these relaxation techniques require regular practice over a period of several weeks, and professional guidance is often necessary in the initial stage of training.

#### Cognitive therapy

Cognitive therapy seeks to alter faulty beliefs and attitudes about sleep.[34] For example, insomniacs "often display a great deal of apprehension about bedtime and performance anxiety in their attempt to control the process of sleep onset; some even entertain catastrophic thinking about the potential consequences of insomnia, all of which may heighten their affective response to poor sleep." The objective of cognitive therapy is to cut short the vicious cycle of insomnia, emotional distress, dysfunctional cognitions, and further sleep disturbances. Examples of treatment targets for cognitive therapy include having unrealistic sleep expectations (e.g., "I must get 8 hours of sleep every night"), misconceptions about the causes of insomnia (e.g., "my insomnia is entirely due to chemical imbalances in my body"), amplifications of its consequences (e.g., "I am going to fail after a poor night's sleep"), and performance anxiety resulting from excessive attempts at controlling the sleep process.[35]

The advocates of cognitive therapy believe that "it consists of identifying patient-specific dysfunctional sleep cognitions, challenging their validity, and replacing them with more adaptive substitutes through the use of restructuring techniques such as reattribution training, decatastrophizing, hypothesis testing, reappraisal, and attention shifting."[36] The evidence for this mode of intervention is the strongest with effect sizes ranging from 0.93 to 1.20 for sleep latency, 0.28 to 0.57 for total sleep time, and 0.28 for wake after sleep onset.[31,32]

#### Paradoxical intention

Paradoxical intention is a method that consists of persuading a patient to engage in his or her most feared behavior, i.e., staying awake.[37] The basic premise is that performance anxiety inhibits sleep onset. Thus, if a patient stops trying to sleep and contrarily attempts to stay awake, performance anxiety will be reduced and sleep may come more easily. This procedure may be conceptualized as a form of cognitive restructuring technique to alleviate performance anxiety. Effect sizes reported have been moderate in sleep latency (0.63–0.73), total sleep time (0.10–0.46), and wake after sleep onset (0.81).[31,32]

#### Sleep hygiene education

Sleep hygiene education targets health practices (e.g., diet, exercise, and substance use) and environmental factors (e.g., light, noise, temperature, and mattress) that may be either detrimental or beneficial to sleep.[38] Although these factors are rarely severe enough to be the primary cause of chronic insomnia, they may complicate an existing sleep problem and hinder treatment progress. Additional recommendations, which tend to overlap with stimulus control and sleep restriction, may also include curtailing daytime napping and time spent in bed. While poor sleepers are generally better informed about sleep hygiene, they also engage in more unhealthy practices than good sleepers. Thus, the objectives of sleep hygiene are to promote better health practices. In a meta-analysis of sleep hygiene, effect size observed was modest in all parameters.[31]

#### Behavioral intervention

Having the patient keep a sleep diary for 2 weeks may be helpful. Depending on the findings in the sleep diary, a discussion of sleep hygiene may be beneficial to the patient. Adopting the practices of good sleep hygiene is often helpful regardless of whether the patient has primary insomnia or a sleep disturbance related to a medical condition.[30] Behavioral psychologists focus on encouraging the patient to eliminate behavior incompatible with sleep, such as lying in bed and worrying, by instructing the patient to leave the bedroom at these times. Patients can condition themselves to be insomniacs, and treatment focuses on de-conditioning the patient from associating the bedroom with a place of restlessness.

#### Summary of non-pharmacologic strategies

There are three recently published meta-analyses which serve to establish the efficacy of psychological and behavioral methods. Pallesen and colleagues[39] evaluated behavioral and psychological interventions in an elderly insomniac patient population (mean age > 60 years). Significant effect sizes in sleep latency (0.64), wake after sleep onset (0.59), and total sleep time (0.37) were observed for psychological/behavioral treatments. Similarly, Irwin and colleagues[40] evaluated treatment efficacy of cognitive behavioral intervention in older adults >55 years old and found significant effects for sleep latency (0.50), wake after sleep onset (0.69), and total sleep time (0.17). However, Montgomery and Dennis[41] reviewed psychological/behavioral and other non-pharmacologic strategies in a similar population and observed minimal changes in sleep latency (mean decrease: 3 minutes) and modest effects on wake after sleep onset (mean decrease: 22 minutes) and total sleep time (mean increase: 14.6 minutes).

### Pharmacologic management strategies

Drug treatment is indicated for patients as short-term alleviation of insomnia but is insufficient for long-term management of chronic insomnia. In combination with behavioral therapy, it however, yields the most durable improvements in sleep patterns.[42]

Although clinical trials of pharmacotherapeutic agents recently approved by the FDA have demonstrated their efficacy and safety, common general practice dictates that five basic principles be followed which characterize rational pharmacotherapy for insomnia, especially chronic insomnia, in both adult and geriatric patients:[43] using the lowest effective dose, using intermittent dosing (two to four times weekly), prescribing medication for short-term use (i.e., regular use for no more than 3–4 weeks), discontinuing the medication gradually, and remaining alert for rebound insomnia following discontinuation.

The therapeutic options include the following.

### First line pharmacotherapy

These drugs carry the highest level of evidence supporting efficacy and safety.

#### Benzodiazepines

Benzodiazepines are frequently prescribed to treat insomnia. These hypnotics reduce latency to sleep onset and total awakenings by increasing total sleep duration. Benzodiazepines enhance the effect of the inhibitory neurotransmitter gamma-amino butyric acid (GABA) by increasing the affinity of GABA for its receptor. Benzodiazepines non-selectively bind to an allosteric site and affect the GABA-A receptor complex to allow a greater number of chloride ions to enter the cell when GABA interacts with the receptor and therefore enhance the inhibitory action of GABA. This accounts for their sedative, anxiolytic, myorelaxant, and anticonvulsant properties. Five benzodiazepines (estazolam, flurazepam, quazepam, temazepam, and triazolam) have an FDA-approved indication for the management of insomnia. Dose, distinguishing pharmacokinetic properties (absorption rate, distribution, and elimination half-life), and risk-benefit ratio should be considered when selecting the most appropriate medication. The lowest effective dose should be used to minimize side effects, and long-acting benzodiazepines with active metabolites should be avoided in the elderly.

Major side effects of short-acting benzodiazepines include rebound insomnia and anterograde amnesia. Intermediate-and longer-acting benzodiazepines are less effective for inducing sleep, but are indicated for sleep maintenance and decreasing nocturnal awakenings.[44] Long-acting medications are best indicated for people with insomnia as well as concomitant daytime anxiety. Accumulation of active metabolites is problematic in elderly patients and in those patients with impaired liver function as it can cause confusion and cognitive dysfunction. Benzodiazepines are contraindicated in patients with acute alcohol intoxication with depressed vital signs, a history of substance abuse, and during pregnancy.

Benzodiazepines should be used cautiously in patients with chronic pulmonary insufficiency or untreated sleep apnea. They are frequently used in mood disorders but a worsening of the dysphoric symptoms and precipitation of suicide has been noted in depression, while hypomania or frank mania and paradoxical hyper-excited states can also occur.[42] However, long-term use (beyond 4 weeks) is associated with dependence, discontinuation syndrome, difficulty in new learning abilities, and blunting of emotions.[45]

#### Non-benzodiazepine hypnotics

Non-benzodiazepine hypnotics include zopiclone, zolpidem, and zaleplon.

#### Zopiclone

Zopiclone is a non-benzodiazepine hypnotic of the cyclopryrrolone class. It is effective for reducing sleep latency and nocturnal awakenings and increasing total sleep time. Zopiclone delays the onset of rapid eye movement (REM) sleep but does not reduce consistently the total duration of (REM) periods. Rebound effects have been reported but are minimal. The incidence of adverse effects is low at recommended doses (3.75–7.5 mg).[46]

#### Zolpidem

Zolpidem is a non-benzodiazepine hypnotic of the imidazopyridine class. It exhibits hypnotic effects with minimal myorelaxant, anticonvulsant, and anxiolytic properties, as it preferentially binds with the GABA-A receptor complexes with an alpha-1 subtype. Zolpidem is effective for reducing sleep latency and nocturnal awakenings and increasing total sleep time. Rebound effects are minimal. Common side effects include drowsiness, dizziness, and headache.[47]

#### Zaleplon

Zaleplon, like zolpidem, belongs to the imidazopyridine class of non-benzodiazepine hypnotics. The pharmacology of these two drugs is similar; however, zaleplon has an ultra-brief duration of effect.[48] It is effective for reducing time to sleep onset, but is not as effective for reducing nighttime awakenings or increasing total sleep time. No next-day sedation or rebound insomnia is documented with zaleplon at recommended doses (5–10 mg).

#### Eszopiclone

Eszopiclone, which is the active stereoisomer of zopiclone, acts as an agonist at benzodiazepine (BNZ) receptors. Well absorbed orally, about 3 mg of eszopiclone is equivalent to 10 mg of diazepam.[49] Although FDA approved for the management of chronic insomnia, there have been several reports of adverse effects like headaches, day-time drowsiness, loss of coordination, GI effects, decreased sexual desire, painful menstruation, and breast enlargement in males, leading a major reviewer to comment that the risk-benefit ratio should be weighed carefully due to the possible adverse effects such as cancer, infection, and death.[50]

#### Ramelteon

A melatonin agonist, it acts by selectively binding to the melatonin receptors (MT1, MT2) in the suprachiasmatic nucleus (SCN). It has also recently been approved for the treatment of insomnia and is the only non-scheduled prescription drug available in the United States for the treatment of insomnia. It has been shown to be effective in the elderly.[51]

No specific agent within this group is recommended as preferable to the others in a general sense; each has been shown to have posi-tive effects on sleep latency, total sleep time (TST), and/or wake after sleep onset (WASO) in placebo-controlled trials.[52-55]

### Second line pharmacotherapy

These drugs have moderate level of evidence supporting their efficacy and tolerability.

#### Antidepressants

*Tricyclic antidepressants* (TCAs) such as amitriptyline, doxepin, and nortriptyline are effective for inducing sleep and improving sleep continuity.[56] These agents should be used at their lowest effective dose to minimize anticholinergic effects and to minimize cardiac conduction prolongation, especially in the elderly. The overdose potential of TCAs is greater than with other hypnotic agents, and daytime sedation can be significant.

#### Trazodone

Trazodone is a potent sedating antidepressant. Trazodone improves sleep continuity and is an attractive option in persons prone to substance abuse, as addiction or tolerance is not a problem.[57] Trazodone is also used in conjunction with stimulating antidepressants such as some SSRIs and bupropion in depressed patients with insomnia. Adrenergic blockade can result in oversedation and orthostatic hypotension, especially in elderly patients. The risk of priapism, a condition of painful, prolonged erection in men, is rare. Other antidepressants used include *Mirtazapine* due to its sedative properties. Evidence for their efficacy when used alone is relatively weak and hence no specific agent within this group is recommended as preferable to the others in this group.[58-61]

#### Antihistamines

Antihistamines are found in many over-the-counter (OTC) sleep aids. These agents are effective for mild insomnia; however, next-day sedation may be a problem. Antihistamines commonly cause psychomotor impairment and anticholinergic effects. Tolerance may also develop with repeated use and evidence for their efficacy and safety is very limited.[62]

### Alternative medications

These are drugs with variable evidence and are useful only in individual cases.

Valerian is a perennial plant that appears to increase GABA concentrations in animal studies, but its exact mechanism is not known. Valerian should not be used for the acute management of insomnia because its hypnotic effect is delayed for 2–4 weeks. Valerian appears to be well tolerated; however, it can cause headache and daytime sedation[63] and is currently still being evaluated.[64]

Other herbs used to promote sleep include skullcap, passion flower, California poppy, and Lemon balm.[65] Melatonin and l-tryptophan are two other molecules undergoing evaluation for the treatment of chronic insomnia.[66] There is currently very little evidence for their use.[67] Indiplon, a novel GABA_A_ potentiator, till recently being studied,[68] has now been abandoned due to its toxicity.

The recommended drugs according to the level of clinical evidence are summarized in Table 3.

**Table 3:** Summary of clinical evidence of pharmacotherapy

Grade A: Highest level of supporting evidence – First line pharmacotherapy		

Agents	Recommended dosage	Comments
Zopiclone	3.75–7.5 mg	Common side effects include drowsiness, dizziness, anterograde amnesia, nightmares, blurred vision, and palpitations
Zaleplon	5–10 mg	Adverse effects include headache, dizziness, somnolence, and nausea
Temazepam/quazepam	10–30 mg	Has the greatest incidence of side effects including dependence and morning after hang over

**Grade B: Moderate level of supporting evidence – Second line pharmacotherapy**		

Amitriptyline	10–50 mg	At low doses, anticholinergic effects rare
Antihistaminics	OTC drugs	Sedation and tolerance are the main problems
Grade C: lowest level of supporting evidence – variable and insuffi cient evidence		
Valerian	400–900 mg	May cause headache and daytime sedation
Ramelteon	8 mg	Approved for chronic insomnia in elderly
Melatonin	1–5 mg	Experimental drugs still being evaluated
l-Tryptophan	0.5–2 g	
Indiplon	10–20 mg	

## Conclusions

The prevalence of chronic insomnia is quite high, yet remains under-diagnosed. It is imperative to recognize it since it may result in increased healthcare utilization, lower quality of life and social relationships, and decrements in memory, mood, and cognitive function. Management strategies favor a combination of cognitive/behavioral therapy and short-term pharmacotherapy with non-benzodiazepine hypnotics being the first line of management. Based on recommendations, an algorithm for treatment is proposed below.

*Step 1:* Evaluate insomnia treatment options (cost, preference, availability).
*Step 2:* Optimize treatment for co-morbid disorder.
*Step 3:* Initiate treatment with cognitive behavior therapy with/without relaxation therapy.
*Step 4:* If no effect, consider another modality of treatment - Type 2.
*Step 5:* If still not improved, consider another modality of treatment - Type 3.
*Step 6:* If still no improvement, re-evaluate especially for occult or co-morbid disorders.
*Step 7:* Reconsider diagnosis.
*Step 8:* Combine with pharmacology - first choice of zaleplon/zopiclone/temazepam/quazepam.
*Step 9:* Combine with pharmacology -second choice of amitriptyline/antihistaminics.
*Step 10:* Try alternative therapies like valerian/ramelteon/melatonin.
